# Orchard Conditions and Fruiting Body Characteristics Drive the Microbiome of the Black Truffle *Tuber aestivum*

**DOI:** 10.3389/fmicb.2019.01437

**Published:** 2019-06-28

**Authors:** Richard Splivallo, Maryam Vahdatzadeh, Jose G. Maciá-Vicente, Virginie Molinier, Martina Peter, Simon Egli, Stéphane Uroz, Francesco Paolocci, Aurélie Deveau

**Affiliations:** ^1^Institute of Molecular Biosciences, Goethe University Frankfurt, Frankfurt, Germany; ^2^Institute of Ecology, Evolution and Diversity, Goethe University Frankfurt, Frankfurt, Germany; ^3^Swiss Federal Research Institute for Forest, Snow and Landscape Research (WSL), Birmensdorf, Switzerland; ^4^UMR 5175 CEFE – CNRS – Université de Montpellier – Université Paul Valéry Montpellier – EPHE, INSERM, Montpellier, France; ^5^Institut National de la Recherche Agronomique, Unité Mixte de Recherche 1136 INRA – Université de Lorraine, Interactions Arbres/Microorganismes, Centre INRA-Grand Est-Nancy, Champenoux, France; ^6^National Research Council (CNR), Institute of Biosciences and Bioresources, Division of Perugia, Perugia, Italy

**Keywords:** *Tuber aestivum*, amplicon sequencing, bacterial communities, microbiome, multilocus genotype, mating type

## Abstract

Truffle fungi are well known for their enticing aromas partially emitted by microbes colonizing truffle fruiting bodies. The identity and diversity of these microbes remain poorly investigated, because few studies have determined truffle-associated bacterial communities while considering only a small number of fruiting bodies. Hence, the factors driving the assembly of truffle microbiomes are yet to be elucidated. Here we investigated the bacterial community structure of more than 50 fruiting bodies of the black truffle *Tuber aestivum* in one French and one Swiss orchard using 16S rRNA gene amplicon high-throughput sequencing. Bacterial communities from truffles collected in both orchards shared their main dominant taxa: while 60% of fruiting bodies were dominated by α-Proteobacteria, in some cases the β-Proteobacteria or the Sphingobacteriia classes were the most abundant, suggesting that specific factors (i.e., truffle maturation and soil properties) shape differently truffle-associated microbiomes. We further attempted to assess the influence in truffle microbiome variation of factors related to collection season, truffle mating type, degree of maturation, and location within the truffle orchards. These factors had differential effects between the two truffle orchards, with season being the strongest predictor of community variation in the French orchard, and spatial location in the Swiss one. Surprisingly, genotype and fruiting body maturation did not have a significant effect on microbial community composition. In summary, our results show, regardless of the geographical location considered, the existence of heterogeneous bacterial communities within *T. aestivum* fruiting bodies that are dominated by three bacterial classes. They also indicate that factors shaping microbial communities within truffle fruiting bodies differ across local conditions.

## Introduction

Truffles are ascomycete ectomycorrhizal fungi that associate with the roots of a large number of trees and shrubs and that produce hypogeous fruiting bodies. Some truffle species such as *Tuber melanosporum* (Périgord black truffle) and *T. aestivum* (Burgundy truffle) are renowned worldwide for their delicate aroma and are considered as culinary delicacies. Although these truffles can be harvested in wild forests, over 80% of the truffles harvested in France are nowadays originating from artificially inoculated orchards (Murat, 2015). In this context, the controlled production of truffles is an economically important goal of research. Major progress has been made over the past 30 years to improve methods of truffle cultivation and to better understand the life cycle of these peculiar fungi (Paolocci et al., 2006; Rubini et al., 2007, 2011a, b; Murat, 2015; Molinier et al., 2016). The most comprehensive knowledge about truffle biology exist about *T. melanosporum* (Rubini et al., 2014; Le Tacon et al., 2016; Selosse et al., 2017) which was the first *Tuber* genome to be sequenced (Martin et al., 2010). However, mounting evidence based on genetics of *T. aestivum* (Molinier et al., 2013a, b, 2016) and the genomics of *T. aestivum* and *T. magnatum* suggests high similarities in terms of life cycle to *T. melanosporum* (Murat et al., 2018). In truffles, the life cycle starts with the germination of haploid spores. Hyphae produced from germinated spores colonize the fine roots of host plants and form ectomycorrhizae. This symbiotic mixed organ is the place of nutrients exchange between the two mutualistic partners (Smith and Read, 2008). Ectomycorrhizae also provide the maternal mycelium that will give birth to the fruiting body (or ascocarp) after mating with a paternal gamete of opposite mating type (Rubini et al., 2014; Selosse et al., 2017, Murat et al., 2018). In contrast to many other ectomycorrhizal fungi that produce fruiting bodies within a few days, the development of truffle fruiting bodies generally takes several months and occurs entirely belowground. In the case of *T. melanosporum*, it has been demonstrated that nutrients required for the development of the fruiting bodies are provided by the host plant all along fruiting body genesis (Le Tacon et al., 2013, 2015) and a similar process likely occurs for *T. aestivum* (Deveau et al., 2019). The production of fruiting bodies in all *Tuber* species varies greatly from year to year, ranging from none to several per tree. Additionally, considering trees with a sufficient degree of mycorrhization with *T. aestivum* or *T. melanosporum*, the yield of harvested truffles was shown to be unrelated to the host tree mycorrhization degree (Molinier et al., 2013a; De la Varga et al., 2017).

Beside the symbiotic association between the fungus and its host, it is now clear that complex microbial communities interact with truffle fungi both in the ectomycorrhizosphere and in the ascocarp. Based on a number of studies on truffle-associated bacterial communities, we know that the surface (peridium) and the inner tissues (gleba) of truffle fruiting bodies are colonized by complex bacterial communities composed of a few hundreds of species that can reach up to 10^7^–10^8^ cells per gram of truffle (Barbieri et al., 2007; Antony-Babu et al., 2014; Vahdatzadeh et al., 2015). The effects of these bacteria and of their interactions on the biology of truffles are still poorly understood. Yet, some bacteria have been shown to participate in the elaboration of some of the volatile organic compounds produced by the whitish truffle *Tuber borchii* (Splivallo et al., 2015), and it has been hypothesized that bacteria could be involved in the elaboration of the complex aroma of truffles (Vahdatzadeh et al., 2015). In addition, some bacteria of the *Bradyrhizobiaceae* family isolated from *T. magnatum* have shown the ability to fix nitrogen (Barbieri et al., 2010). It has been proposed that they could participate in the nutrition of the fungus during fruiting body development (Barbieri et al., 2010). Additional putative effects such as inhibition of pathogenic fungi, stimulation of the growth of *Tuber* mycelium, and ascocarp degradation have also been suggested based on potential functional activities of bacteria isolated from fruiting bodies (Citterio et al., 2001; Sbrana et al., 2002; Dominguez et al., 2012; Gryndler et al., 2013, 2015; Antony-Babu et al., 2014; Saidi et al., 2015; Deveau et al., 2016).

Despite differences between truffle species (Benucci and Bonito, 2016), the truffle microbiome is commonly dominated by bacteria belonging to the *Rhizobiales* order together with, to a lesser extent, members of the orders Actinomycetales, Burkholderiales, Enterobacteriales, Flavobacteriales, and Pseudomonadales (Barbieri et al., 2016). Yet, important variations in the composition of truffle microbiomes have been reported (Barbieri et al., 2016). Part of the discrepancies may be explained by the evolution of methodologies used to study microbial diversity, which cover from culture-dependent to various generations of culture-free methodologies (Sbrana et al., 2002; Barbieri et al., 2010; Deveau et al., 2016). Another part of this variability could be due to natural variation in microbiome composition among fruiting bodies of single *Tuber* species. Among the different factors that could influence truffle microbiome composition, the level of fruiting body maturation has been proposed as a potential driver of the microbiome composition in *T. borchii*, *T. indicum*, and *T. melanosporum* (Citterio et al., 2001; Antony-Babu et al., 2014; Splivallo et al., 2015; Ye et al., 2018). However, the extent to which other intrinsic (i.e., maturity, genotype, mating type) and extrinsic (i.e., season, location, spatial distance) factors drive the truffle microbiome is not known.

In this study, we filled this gap in knowledge by analyzing and comparing the microbiomes of more than 50 fruiting bodies of *T. aestivum* harvested over several years in two spatially distant orchards in Europe. *T. aestivum* is harvested and cultivated in numerous regions of the world (i.e., all over Europe, in Iran, Northern Africa) and its microbiome has not been extensively studied despite the fact that it represents one of the most relevant truffles in terms of traded volumes. We hypothesized that the microbial communities of *T. aestivum* would be dominated by bacteria of the *Bradyrhizobiaceae* family as in other truffle species but also that noticeable differences in microbial assemblages would be detectable between the two study sites due to variable environmental factors. To test and answer those hypotheses, (1) the “core” composition of the *T. aestivum* microbiome in both study sites was defined, (2) the variability in the truffle microbiome across orchards was assessed, and (3) the intrinsic factors (maturity, genotype, mating type) and extrinsic ones (season, location, spatial distance) determining the assembly of truffle-associated bacterial communities were evaluated.

## Materials and Methods

### Biological Material and Sampling

Fruiting bodies of *Tuber aestivum* (Vittad.) were collected from two artificially inoculated truffle orchards in France (FR) and Switzerland (SW). Exact GPS coordinates are not given here at the request of the orchard’s owners, but the closest city nearby is provided as an approximate location. These orchards have been described earlier (Splivallo et al., 2012; Molinier et al., 2013a, 2015). The French orchard, located near Daix/Dijon (FR), is a 30-year old truffle orchard that comprises two rows of inoculated hazels (*Corylus avellana*) at its center and two outer rows of fruit trees on the outer margins (see for details Molinier et al., 2013a). All hazel trees in the French orchard were inoculated with *T. melanosporum* in 1976 and produced *T. melanosporum* fruiting bodies for nine seasons from 1980 to 1989 (a few hundred grams to 12 kg per year; Molinier et al., 2013a). During 1990–1993 production was gradually and eventually fully replaced by native *T. aestivum* and in subsequent years, production ranged from a few hundred grams to a few kilograms of *T. aestivum* per year (Molinier et al., 2013a). The soil of the French truffle orchard has a calcareous nature and a pH of 7.9 (Molinier et al., 2013a). The Swiss orchard, located in Valais, near St-Triphon (CH), contains 42 trees – oaks (*Quercus robur*) and pines (*Pinus nigra*) that were commercially inoculated with *T. aestivum* and planted in 1999. In this orchard, pH of the soil is 7.6 and production of *T. aestivum* started in 2008/2009 and ranged since then to a few hundred grams to approximately 1 kg per year.

**FIGURE 1:**
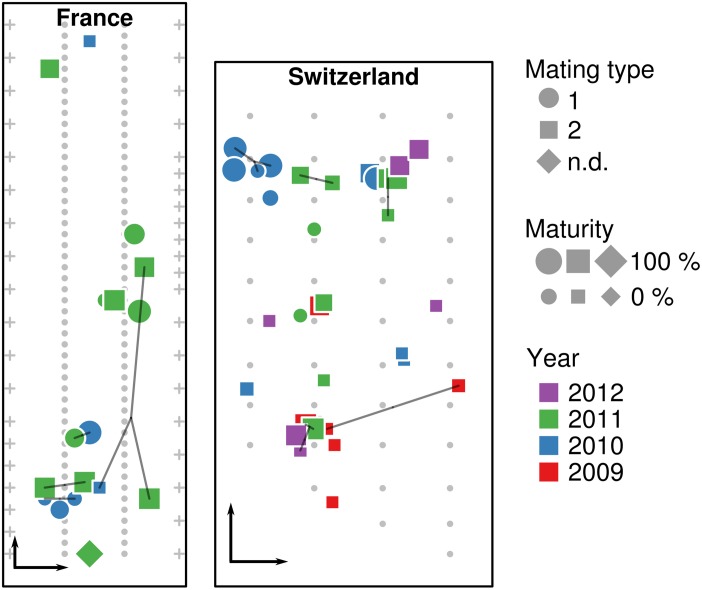
Location of truffle samples within the French and Swiss orchards. Location of truffles, their maturity, mating type, collection year, and identical multilocus genotypes (MLGs, connected by lines) are shown in the French and Swiss orchards, along the position of truffle-mycorrhized trees (small gray dots) and fruit trees surrounding the orchard (crosses). Black arrows in the lower left corner of each orchard represent a distance of 5 m.

A total of 62 *T. aestivum* fruiting bodies were collected from the two artificially inoculated truffle orchards. Seventeen truffles were collected in the French orchard in 2010 and 2011, whereas 45 truffles were collected from the Swiss orchard during four consecutive years (2009–2012). The precise location of truffles was recorded at the time of the harvest (Figure 1). To avoid post-harvest drifts of microbial populations, all truffles were cooled to 4°C after collection and frozen to −20°C within 24 h for subsequent DNA extraction.

Truffles were identified by spore morphology and via molecular methods (see the section “DNA Extraction and Truffle Genotyping”). The stage of fruiting body maturation was determined by estimating the percentage of ascii containing melanized spores, as previously described (Splivallo et al., 2012). An overview of the samples used in this study along with the analyses performed is shown in Table 1.

**TABLE 1:** Details of the *Tuber aestivum* samples originating from the Swiss and French orchard, and results of bacterial microbiome analysis.

**Microbiome analysis**											
**Sample code**	**Collection year**	**Collection month**	**Maturity (0% = fully immature, 100% = fully mature)**	Genotype (MLG)^*^ CH = Swuitzerland FR = France	**Mating type**	**Sequencing technique (454 or MiSeq)**	**Number of sequencing reads before processing**	**Number of reads post processing**	**Number of OTU^†^**	**Shannon**	**Effective number of species**
13	2009	October	0%	CH_27	2	454	15,204	7,149	26	0.288	1
14	2009	October	0%	CH_29	2	454	17,689	7,058	8	0.394	1
15	2009	October	0%	CH_27	2	n/a	n/a	n/a	n/a	n/a	n/a
17	2009	October	0%	CH_22	2	454	27,548	12,868	26	0.672	2
18	2009	October	95%	CH_22	2	n/a	n/a	n/a	n/a	n/a	n/a
19	2009	October	11%	CH_22	2	454	17,645	8,280	65	2.075	8
20	2009	October	0%	CH_28	2	454	26,269	11,859	6	0.268	1
21	2009	October	0%	CH_27	2	n/a	n/a	n/a	n/a	n/a	n/a
24	2009	December	93%	CH_5	2	454	9,643	4,561	45	0.648	2
25	2009	December	54%	n/a	2	454	44,804	22,909	27	0.196	1
26	2009	December	73%	CH_17	2	454	22,250	10,740	53	1.350	4
27	2010	September	92%	CH_33	1	454	18,690	8,894	77	2.403	11
28	2010	September	2%	CH_31	1	n/a	n/a	n/a	n/a	n/a	n/a
29	2010	September	0%	CH_3	2	454	15,096	7,390	26	0.204	1
30	2010	September	87%	CH_34	1	454	27,672	12,367	17	1.061	3
31	2010	September	1%	CH_34	1	454	19,699	9,673	23	0.862	2
32	2010	September	84%	CH_8	1	454	14,311	6,821	4	0.679	2
34	2010	September	82%	CH_7	1	454	24,439	10,966	9	0.548	2
40	2010	September	15%	CH_20	2	454	10,840	4,965	34	0.886	2
43	2010	September	69%	CH_11	2	454	9,640	4,303	14	0.071	1
44	2010	December	96%	CH_34	1	454	32,011	14,956	6	0.750	2
45	2010	December	0%	CH_2	2	454	24,123	11,645	54	0.755	2
47	2010	December	13%	CH_31	1	n/a	n/a	n/a	n/a	n/a	n/a
48	2010	December	19%	CH_32	1	454	26,987	13,060	5	0.140	1
49	2010	December	0%	CH_25	1	n/a	n/a	n/a	n/a	n/a	n/a
50	2010	December	25%	CH_16	1	n/a	n/a	n/a	n/a	n/a	n/a
51	2010	December	85%	CH_19	1	n/a	n/a	n/a	n/a	n/a	n/a
52	2010	December	86%	CH_24	2	n/a	n/a	n/a	n/a	n/a	n/a
53	2011	August	55%	CH_18	2	454	28,556	13,665	9	0.023	1
55	2011	August	30%	CH_18	2	454	12,310	6,054	56	2.300	10
57	2011	August	0%	CH_13	2	454	16,204	8,140	12	0.062	1
59	2011	August	0%	CH_30	2	454	22,247	11,161	9	0.029	1
63	2011	November	70%	CH_9	2	454	14,862	6,325	9	0.329	1
65	2011	November	5%	CH_9	2	454	31,539	15,919	6	0.055	1
67	2011	November	0%	CH_23	1	454	27,997	13,450	7	0.249	1
69	2011	November	95%	CH_21	2	454	18,199	9,109	7	0.024	1
71	2011	November	95%	CH_5	2	454	18,022	9,271	37	0.340	1
73	2011	November	50%	CH_15	2	454	20,369	9,762	12	0.042	1
75	2011	November	0%	CH_14	1	454	15,607	7,883	9	0.070	1
77	2012	August	75%	CH_12	2	454	19,118	9,791	22	0.158	1
78	2012	August	75%	CH_10	2	454	14,444	6,658	24	1.816	6
79	2012	August	5%	CH_27	2	454	24,087	12,414	28	0.236	1
80	2012	December	1%	CH_26	2	454	24,658	12,032	15	0.101	1
82	2012	December	85%	CH_6	2	454	21,038	9,811	13	0.052	1
84	2012	December	0%	CH_1	2	454	23,360	10,788	16	0.599	2
D1	2010	October	0%	FR_23	n/a	MiSeq	33,632	26,296	97	2.613	13
D2	2010	October	7%	FR_23	1	MiSeq	44,386	41,798	91	1.300	4
D3	2010	October	44%	FR_2	1	MiSeq	46,082	43,159	109	1.213	3
D4	2010	October	0%	FR_12	2	MiSeq	37,830	33,175	83	1.484	4
D5	2010	October	0%	FR_10	2	MiSeq	37,405	36,450	75	0.401	2
D21	2010	December	94%	FR_20	n/a	MiSeq	37,956	35,472	57	2.113	8
D25	2011	October	0%	FR_22	1	MiSeq	38,708	38,159	53	0.178	1
D26	2011	October	70%	FR_15	2	MiSeq	37,358	36,499	55	0.301	1
D27	2011	October	90%	FR_6	2	MiSeq	40,495	35,131	96	1.700	6
D28	2011	October	88%	FR_18	1	MiSeq	40,245	38,394	68	0.615	2
D29	2011	October	92%	FR_12	2	MiSeq	39,319	38,719	49	0.224	1
D30	2011	October	50%	FR_20	1	MiSeq	39,187	38,520	37	0.086	1
D31	2011	October	87%	FR_8	2	MiSeq	38,060	36,839	34	0.644	2
D32	2011	October	91%	FR_8	2	MiSeq	39,274	38,597	36	0.194	1
D34	2011	October	65%	FR_5	1	MiSeq	31,638	28,588	60	2.075	8
D35	2011	November	81%	FR_4	n/a	MiSeq	39,007	37,880	69	0.302	1
D36	2011	November	82%	FR_12	n/a	MiSeq	40,582	39,764	46	0.167	1

^*^MLGs are named as CH for Switzerland and FR for France followed by a number. Note that the same number (i.e., CH_5 and FR_5) do not refer to the same MLG as the MLG analysis for both orchards were done independently from each other. ^†^Number of OTUs after singleton removal and elimination of rare OTUs (OTUs with a total number of reads inferior to 0.01% of the total number of all samples). n/a, missing data.

### DNA Extraction and Truffles Genotyping

Genomic DNA was extracted from the gleba (50–100 mg fresh weight excised from the central part of the gleba) of each fruiting body using the DNeasy^®^ Plant Mini Kit (Qiagen, Hilden, Germany) following the manufacturer’s instructions. Even though this kit might have been used here for characterizing truffle’s microbiome for the first time, earlier works have demonstrated that various DNA extraction methods yielded comparable microbiome compositions for different truffle species (Antony-Babu et al., 2013; Benucci and Bonito, 2016). DNA qualities and concentrations were checked using a NanoDrop spectrometer and gel electrophoresis. Mating type identification was performed using the specific primers aest-MAT1-1f/aest-MAT1-1r and aest-MAT1-2f/aest-MAT1-2r as described elsewhere via multiplex polymerase chain reaction (PCR) (Molinier et al., 2016). In short, PCRs were carried out using 3 μl of template DNA (diluted 10 times) in a final volume of 20 μl containing 10 μl of JumpStart REDTaq ReadyMix (Sigma-Aldrich: P1107), 0.4 μl of each primer (0.2 μM each), and water to adjust to the final volume. Thermal cycles were conducted using the following program: an initial denaturation of 2 min at 94°C, 28 cycles at 94°C for 30 s, 57°C for 30 s, and 72°C for 1 min, followed by 72°C for 7 min. PCR products were checked on a 1.5% agarose gel and visualized after ethidium bromide staining by a UV transilluminator.

A total number of 14 SSR loci (aest01, aest06, aest07, aest10, aest15, aest18, aest24, aest25, aest26, aest28, aest29, aest31, aest35, and aest36) (Molinier et al., 2013b) were chosen for genotyping. The genotyping procedure followed that described by Molinier et al. (2016) but with a slightly modified PCR program: one cycle of 15 min at 95°C, 30 cycles of 30 s at 94°C, 90 s at 60°C, and 60 s at 72°C, and a final elongation cycle of 30 min at 60°C. To identify multilocus genotypes (MLGs) and true clones based on the 14 SSR markers, the software MLG_SIM_ (Stenberg et al., 2003) was used as described elsewhere (Molinier et al., 2016).

### Microbiome Analysis

Bacterial communities of Swiss fruiting bodies were analyzed by 454 pyrosequencing, while French samples were analyzed by MiSeq Illumina sequencing, because 454 pyrosequencing technique was no longer available at the time of the analysis. In both cases, the isolated DNA from the gleba of fruiting bodies was used to generate 16S rRNA gene amplicon libraries using the primers 787r (ATTAGATACCYTGTAGTCC) (Nadkarni et al., 2002) and 1073F (ACGAGCTGACGACARCCATG) (On et al., 1998), modified to include specific linkers and identification barcode sequences for the respective sequencing method. The same procedure as described by Antony-Babu et al. (2014) was used to generate 454 pyrosequencing amplicons. Briefly, the PCRs contained 10 μl of PCR Mastermix (5 PRIME), 1 μl of each forward and reverse primers (each 0.2 μM), and 2 μl of template DNA (or sterile water for negative control) in a final volume of 25 μl. For each truffle DNA sample, amplifications were performed in three parallel PCR tubes under the following conditions: an initial denaturation at 94°C for 10 min followed by 30 cycles of denaturation at 94°C for 30 s, annealing at 48°C for 45 s, extension at 72°C for 90 s, and a final extension at 72°C for 10 min. The three PCR products were pooled and quantified by gel electrophoresis and an equimolar mix of amplicons was used for pyrosequencing. Amplicon sequencing was performed by the GS-FLX 454 Titanium platform of Beckman Coulter Genomics (Danvers, MA, United States). Illumina MiSeq amplicons were produced using the same amplification protocol except that the identification barcode sequences were added through a second round of amplification as described by Barret et al. (2015). PCR cycling conditions were 94°C for 2 min, followed by 12 cycles of amplification at 94°C for 1 min, 55°C for 1 min, and 68°C for 1 min each, and a final extension step at 68°C for 10 min. All amplicons were purified with the Agincourt AMPure XP system and quantified with QuantIT PicoGreen. The purified amplicons were then pooled in equimolar concentrations, and the final concentration of the library was determined using a quantitative PCR (qPCR) next-generation sequencing (NGS) library quantification kit (Kapa Biosystems, Boston, MA, United States). Amplicon libraries were mixed with 10% PhiX control according to the 2 × 250 bp Illumina protocols. The second round of PCRs, the purification steps, and sequencing was performed by the GeT PLAGE sequencing platform according to standard procedures (INRA Toulouse). The standard procedure to generate libraries for Illumina Miseq is available here: https://support.illumina.com/documents/documentation/chemistry_documentation/16s/16s-metagenomic-library-prep-guide-15044223-b.pdf.

Both 454 pyrosequencing and MiSeq Illumina 16S rRNA sequences were analyzed using FROGS (Escudié et al., 2018). After quality control and demultiplexing, sequences were preprocessed by removing primers from sequences, sequences out of the amplicon size range (250–300 bp), sequences with only one primer, with at least one homopolymer longer than 7 bp and a Phred quality score <10, and replicates of identical sequences. For the MiSeq Illumina run, 16S rRNA paired-end sequences were first merged (289 bp). Sequences were clustered into operational taxonomic units (OTUs) at 97% sequence similarity based on the iterative Swarm algorithm, with subsequent removal of chimeras for further analysis. Taxonomy assignment to each cluster was carried out by BLAST comparisons against the SILVA database and using the RDP Classifier (Ribosomal Database Project; Cole et al., 2009). OTUs with poor affiliation or higher abundance in negative controls than samples were deleted for further analysis. Finally, OTUs with a total number of reads inferior to 0.01% of the total number of all samples were discarded. The raw data are deposited in the NCBI Sequence Read Archive website^1^ under the BioProject study accession number PRJNA506316 and the SRA accession numbers SRX5059925–SRX5059959 (454 sequences) and SRX5073276–SRX5073292 (Illumina sequences).

### Statistical Analyses

All statistical analyses were performed in R (R Core Team, 2017) with the aid of relevant packages. The datasets from FR and SW were processed independently, but using the same procedure. The datasets were only combined to generate a joint heat tree using the R package Metacoder v0.1.3 (Foster et al., 2017), to summarize the overall taxonomic composition obtained and compare the relative proportion of taxa between both studies. Differences between sites in the relative abundances of the main bacterial taxa were assessed via one-way ANOVA followed by Tukey’s HSD *post hoc* test, after verifying normality of data with Shapiro–Wilk test. Overall and per-sample rarefaction curves were calculated in each dataset to assess sampling completeness, using function *rarecurve()* in package Vegan v3.5-1 (Oksanen et al., 2015). Based on these, subsequent analyses of diversity and community structure were performed on datasets where samples had been rarefied with the Phyloseq package (McMurdie and Holmes, 2013) to achieve equal read numbers of 26,295 for the FR dataset and 3,855 for the SW dataset. Rarefaction curves were used to verify that the subsampling was performed as close as possible to the asymptotes to allow comparison between samples in both French and Swiss sites (Supplementary Figure 1). Values of OTU richness and diversity based on Shannon’s index were calculated using functions available in Vegan. Effective numbers of species were calculated using Simpson index as proposed by Jost (2006).

Non-metric multidimensional scaling (NMDS) was used to visualize differences in community composition among samples. NMDS is an ordination method that represents pairwise (dis)similarities between samples in a low-dimensional space, so that samples placed closer in the graph are more similar than those further apart (Clarke, 1993; Legendre and Legendre, 2012). NMDSs were based on Bray–Curtis dissimilarities calculated among samples after a Hellinger transformation of data (Legendre and Gallagher, 2001).

We investigated the potential influence of factors on microbiome variation using variation partitioning based on distance-based redundancy analysis (db-RDA; Borcard et al., 2011; Legendre and Legendre, 2012), with the Hellinger-transformed dissimilarity matrix as response variable. db-RDA is a constrained ordination method in which a matrix of pairwise (dis)similarities between samples is modeled as a function of a set of explanatory variables (Legendre and Legendre, 2012). Variation partitioning can then be applied to measure the relative contribution of individual explanatory variables to overall community variation while accounting for the effects of other variables, by sequentially removing components from the db-RDA model and recording the resulting changes in the total variance explained.

The explanatory variables to be included in the variation partitioning analysis were selected, when possible, by means of permutational analysis of variance (PERMANOVA; Anderson, 2001), so that only those with a significant correlation with community variation (*P* < 0.05) in at least one dataset were retained. The potential influence on community structure of spatial distance among samples was first examined using Mantel correlograms (Legendre and Legendre, 2012), which enable to test whether samples that are spatially close are more similar than those farther apart. Then, to allow their inclusion in the variation partitioning analysis, the spatial relationships were summarized as principal coordinates of neighbor matrices (PCNM) vectors (Legendre and Legendre, 2012), calculated from the coordinates of each sample within the orchard using the function *pcnm()* of vegan. PCNMs describe non-random patterns in dissimilarity matrices at different scales, which can then be used to model potential sources or variation not accounted for by the measured explanatory variables, such as dispersal, species interactions, or historical causes (Peres-Neto and Legendre, 2010). PCNM vectors significantly associated with community variation in our datasets were forward-selected using package Packfor v0.0-8 (Dray et al., 2009). Because no PCNMs were significantly correlated with the FR dataset, in this case, we manually selected the first four to match the number of PCNMs retained for SW. As done with spatial distances among samples, the factor truffle genotype was assessed by summarizing Euclidean distances among SSR profiles with PCNM vectors and testing their association with microbiome variation by forward selection. After the selection of factors, the final db-RDA models included as explanatory variables truffle mating type, degree of maturity, year of collection, and spatial distance. Truffle maturity and SSR genotypes were excluded because they did not explain an important nor significant amount of microbiome variation in any location.

## Results

### High-Throughput Sequencing

A total of 661,164 and 757,177 quality-passed sequences were obtained for the French (Illumina sequencing) and Swiss (454 sequencing) orchards, respectively, with averages of 38,892 (±807 SE) and 21,033 (±1,209) reads per sample (Table 1). After quality filtering and removal of chimeric reads, a total of 623,440 and 362,697 sequences were retained, with an average of 36,673 (±1,023) and 10,075 (±610) reads per sample across the French and Swiss samples, respectively (Table 1). After taxonomy assignment, elimination of contaminants and of OTUs present in <0.01% of the total number of reads (128 and 1,177 OTUs, respectively), 183 and 147 OTUs were considered for further analyses in the French and Swiss samples, respectively.

### Truffles From Two Distant Orchards Have Similar Microbiomes

**FIGURE 2:**
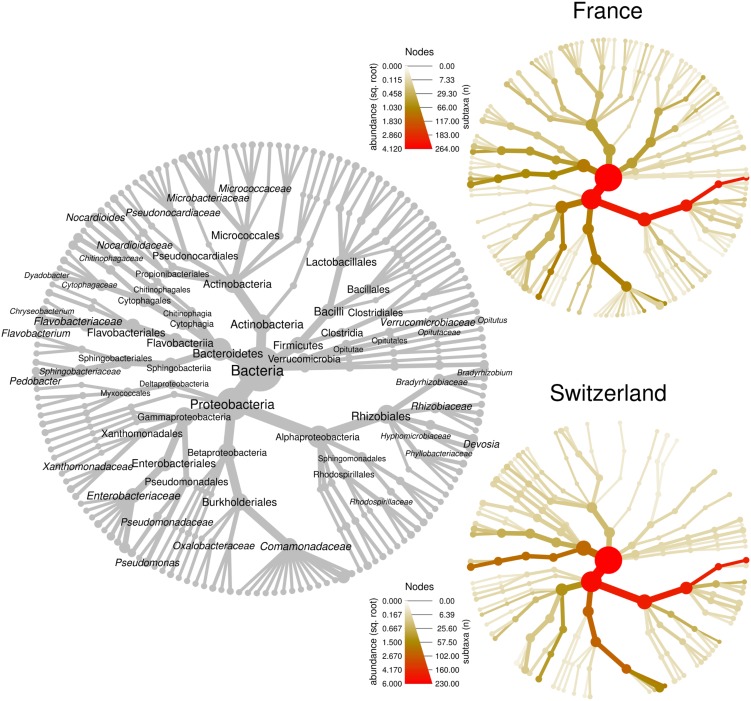
Bacterial community structure within *T. aestivum* fruiting bodies collected from two orchards. Each heat tree represents bacterial community structure as a taxonomic hierarchy up to genus level. The gray tree serves as a key for the smaller unlabeled trees, node labels highlight the most abundant taxa detected at both orchards. The smaller colored trees show community structure for each orchard, assessed with different amplicon sequencing technologies [MiSeq for France (FR), 454 for Switzerland (SW)]. Node and edge sizes are proportional to the number of OTUs within each taxon, whereas color represents taxon abundances (square root of read numbers).

We first compared the microbiome composition of truffles collected from the French and Swiss orchards. An average of 66 ± 6 (SE) OTUs were detected in the French samples, while 23 ± 3 were recorded in average in the Swiss ones. This important difference could be a bias due to the use of MiSeq Illumina sequencing (French orchard) and 454 pyrosequencing (Swiss orchard). Indeed, Illumina sequencing allows for larger numbers of reads per sample and may provide a better access to rare OTUs. This hypothesis was confirmed on another set of data obtained from *T. melanosporum* fruiting bodies analyzed both by 454 and MiSeq Illumina sequencing (Deveau et al., unpublished data). The two samples that were analyzed by both methods strongly differed in richness (21 vs. 98 OTUs for 454 and Illumina MiSeq, respectively) but the relative abundance of the dominant genera that were found in this study was similar no matter which methodology was used (Supplementary Figure 2). In accordance with this observation and despite the two different sequencing methods used, the general composition of the truffle microbiomes detected at each site was alike, as shown in Figure 2. In both locations, communities were dominated by OTUs affiliated to the α-Proteobacteria (FR: 67 ± 9% SE, CH: 63 ± 7%; *P* > 0.05) followed by closely related proportions of Bacteroidetes (FR: 9 ± 4%; CH: 14 ± 5%; *P* > 0.05), β-Proteobacteria (FR: 9 ± 6%, CH: 17 ± 5%; *P* > 0.05), and γ-Proteobacteria (FR: 10 ± 4%, CH: 4 ± 2%; *P* > 0.05). Overall, Actinobacteria (FR: 2.5 ± 0.7%, CH: 0.6 ± 0.2%; *P* < 0.01) and Firmicutes (FR: 2.7 ± 1.7%, CH: 0.1 ± 0.02%; *P* > 0.05) were less frequent, with Actinobacteria being the only phylum with a significantly different abundance between the two orchards. OTUs of d-Proteobacteria, Acidobacteria, and Verrucomicrobia were found at very low levels in some samples of the two sites. Strong similarities between the two orchard’s samples were also observed at the genus level, since the most represented OTUs belonged to the same genera: *Bradyrhizobium* (FR: 65.1 ± 8.8%, CH: 58.6 ± 6.9%), *Pseudomonas* (FR: 8.1 ± 3.4%, CH: 3.4 ± 1.4%)*, Pedobacter* (FR: 4.3 ± 3.3%, CH: 13.8 ± 4.9%), *Polaromonas* (FR: 5.4 ± 5.0%, CH: 9.2 ± 4.4%), and *Flavobacterium* (FR: 2.5 ± 1.2%, CH: 0.8 ± 0.7%). Twenty-three additional genera were shared between the two datasets. This “core” microbiome contained genera belonging to five different Phyla (Supplementary Table 1). Differences nevertheless also existed between the two localities at the genera level as several dozens of genera were also specifically found on one of the two orchards. Yet it is here difficult to discriminate the part of sequencing methodology bias from true data.

### α-, β-Proteobacteria, and Sphingobacteriia Dominate Single Fruiting Bodies

**FIGURE 3:**
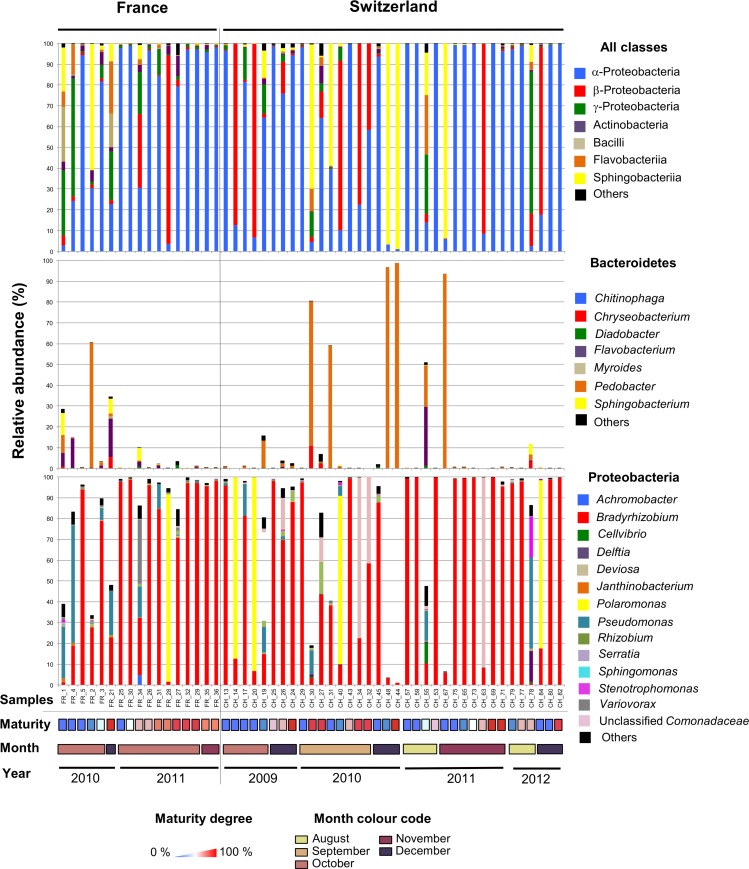
The microbiome of *T. aestivum*. Distribution of major bacterial classes (top panel) and genera (intermediate and bottom panels) in the French and Swiss truffle fruiting bodies analyzed here. Each column represents a single sample. For each sample, three pile-up plots are given: the relative distribution of reads among the major bacterial classes (top panel) and of the different genera forms the Bacteroidetes (intermediate panel) and Proteobacteria phyla (bottom panel). Samples were ordered according to the year and month of collection, and maturity degree (from low to high).

Having compared the overall microbiomes of the French and Swiss orchards, our next aim was to assess the variability in bacterial community structure and taxonomic composition among fruiting bodies. The bacterial community structure and composition was highly variable among single fruiting bodies collected within the same orchard. In both the French and Swiss orchards, the number of OTUs detected per truffle samples varied from a few OTUs to more than a hundred (Table 1). Such variation was independent from the sequencing depth obtained for each sample (Table 1). It is thus likely not due to a bias of sequencing depth but rather reflects different patterns of bacterial community structures, some truffles being colonized by a small number of species while others harbored a larger number of species. The evenness of the bacterial communities also deeply differed between samples in both orchards as illustrated by the strong variability of the Shannon and the effective species value (Table 1). While most truffle-associated bacterial communities were dominated by a few abundant OTUs and numerous rare OTUs, a few fruiting bodies of both sites showed more balanced patterns (data not shown). Such heterogeneity was also reflected when looking at the composition of the bacterial communities at different taxonomic levels (Figure 3). Overall, at the phylum level, 57% of the fruiting bodies showed communities dominated by α-Proteobacteria while 13% of the fruiting body communities were dominated by β-Proteobacteria, and 11% by Bacteroidetes. Eight percent of the fruiting bodies harbored balanced communities in which several phyla were co-dominants. A similar pattern was maintained at the genus level, with *Bradyrhizobium* (α-Proteobacteria), *Polaromonas* (β-Proteobacteria), and *Pedobacter* (Sphingobacteriia) being the most abundant genera depending on the fruiting body considered. To a lesser extent, OTUs from the *Variovorax* genus (β-Proteobacteria), *Pseudomonas* (g-Proteobacteria), *Sphingomonas* (α-Proteobacteria), and *Flavobacterium* (Flavobacteria) formed a significant part of the communities in some fruiting bodies. Thus, the large sampling effort realized over several years in the two truffle orchards revealed the existence of an unsuspected important variability in the composition of the microbiome of truffle ascocarps.

### Mating Type and Multilocus Genotype Distribution of Truffle Fruiting Bodies Within the Orchards

Truffle fruiting bodies result from the fertilization of two individuals of opposite mating type (Martin et al., 2010; Rubini et al., 2011b). Whereas the truffle gleba (maternal tissue) is made up by one individual, the spores contain meiotic products of the two mating partners (Paolocci et al., 2006; Rubini et al., 2011b; Selosse et al., 2017). Here, we determined the genetic profile of the truffle gleba (maternal genotype) only, since the gleba harbors most of the truffle microbiome (Antony-Babu et al., 2014; Splivallo et al., 2015).

Genotyping of the truffles from the French orchard had been done in a previous study (Molinier et al., 2016). A large proportion of unique genotypes (i.e., genotypes that were recorded only once) was observed: eight truffles had unique genotypes and only two pairs with the same MLG (here FR12 and FR20) were detected over the 2010–2011 seasons (Table 1). Truffles of opposite mating types appeared to be evenly spread over the French orchard. In the Swiss orchard, out of the 44 fruiting bodies for which the MLG was identified, 26 had unique MLGs, whereas other MLGs were shared among the remaining 18 samples. In particular, four MLGs (namely CH_5, CH_9, CH_18, and CH_31) were shared between two individuals, two (CH_22 and CH_34) among three individuals, and only one (CH_27) among four individuals (Figure 1). In terms of collection season, five out of seven shared MLGs were found in truffles harvested in the same season, while only two MLGs were shared by truffles harvested 2–3 years apart. Last and by contrast to the French orchard, truffles of mating type 1 were strongly aggregated in one corner of the Swiss field, whereas the rest of the orchard was dominated by truffles of mating type 2 (Figure 1).

### Different Factors Affect Truffle’s Microbial Communities in the French and Swiss Orchards

Having observed important differences in microbial community composition and structure within truffles of the same orchard, we explored whether this variability could be linked to a series of biotic and abiotic factors inherent to truffle ascocarps and to truffle orchards. Specifically, we considered seasonality, space (the location of truffles within an orchard), fruiting body genotype, mating type, and maturation as potential factors affecting the microbiome.

**FIGURE 4:**
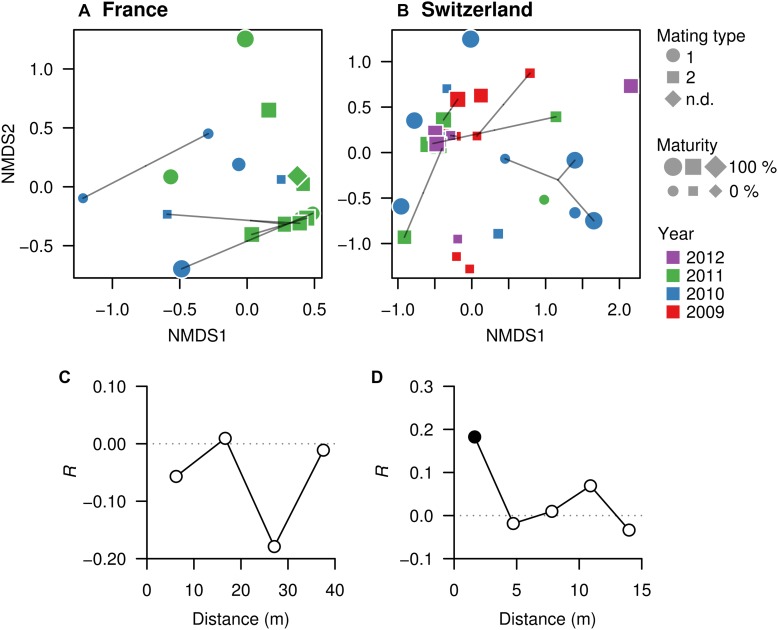
Microbial community similarities among truffles within orchards. (**A**, FR; **B**, SW) Non-metric multidimensional scaling (NMDS) ordinations based on Bray–Curtis dissimilarities of the OTU composition of microbiomes in truffle samples. The closer the samples, the more similar their microbiomes are. Different symbols denote mating type, symbol sizes represent maturity of fruiting bodies, and symbol colors represent collection year. Points linked with lines are fruiting bodies belonging to the same MLG. (**C**, FR; **D**, SW) Correlograms showing correlation of microbiome similarity among samples (*y*-axis, Mantel’s *R*) with spatial distance (*x*-axis). Solid and empty points denote significant (*P* < 0.05) and non-significant correlations for each distance class based on Bonferroni adjustment for multiple testing, indicating that space affects the truffles microbiomes in the Swiss but not the French site.

Non-metric multidimensional scaling was used to visualize pairwise dissimilarities between each truffle-associated microbiome and to explore their relationships with intrinsic (maturation, genotype, mating type) and extrinsic factors (collection season, year, or spatial distance) potentially explaining microbial community structure. NMDS ordinations based on Bray–Curtis dissimilarities among samples in each field showed no evident sample groupings related to truffle maturity, mating type, or MLGs (Figures 4A,B). However, in the Swiss orchard, mating type was significantly associated with microbiome variation based on PERMANOVA analysis (Figure 4B; *F_(_*_1,35__)_ = 4.6, Adj. *R*^2^ = 0.12, *P* < 0.002), whereas in the French field, a significant effect was found for the collecting year (*F*_(__1,16__)_ = 3.2, Adj. *R*^2^ = 0.17, *P* = 0.016). Likewise, a different effect of spatial distance on bacterial communities was found in each site: whereas no association was found in the French orchard (Figure 4C), in SW, similarities among truffle microbial communities appeared to be significantly influenced by distance, with a strong aggregation pattern up to a distance of approximately 2 m (Figure 4D).

Distance-based redundancy analysis ordinations were applied to model the variation in truffle microbiomes in the French and Swiss orchards as a function of the explanatory variables that significantly influenced the microbiome: truffle mating type, year of collection, and spatial distribution of samples (PCNMs). Other factors (maturation and genotype) were excluded as they did not have a significant effect as demonstrated earlier (see also the section “Materials and Methods”). The db-RDAs models constrained by truffle mating type, year of collection, and spatial distribution explained 19% (*F*_2_ = 2.3, *P* = 0.002) and 18% (*F*_2_ = 1.5, *P* = 0.048) of overall community variation for the Swiss and French orchards, respectively. In the Swiss orchard, the associations of community structure with mating type and spatial factors previously reported were confirmed (Figure 4B), with spatial distance and mating type explaining an overall 18 and 9% of total variation, respectively, after accounting for the effects of other variables (Table 2). These values contrasted with a comparably low contribution (1.0%) of collection year (Table 2). In FR, the only variable with a significant correlation with microbiome structure was the collection year, with an overall 11% (*P* = 0.010) of the variance explained (Table 2).

**TABLE 2:** Variation in microbial community composition explained by mating, year of collection, and space.

	**France**			**Switzerland**		
	***df***	**Percentage of variation explained (Adj. *R*-square)**	***P***	***df***	**Percentage of variation explained (Adj. *R*-square)**	***P***
Mating	1	0.03	0.154	1	**0.09**	**0.001**
Year	1	**0.11**	**0.010**	1	0.01	0.169
Space	4	−0.09	0.872	4	**0.18**	**0.001**
Residuals	n.d.	0.82	n.d.	n.d.	0.81	n.d.

*For each orchard (FR, SW), the table lists the proportion of overall variation exclusively explained by each factor based on variation partitioning analysis of db-RDA models. Significant values (*P* < 0.05) are shown in bold typeface. The factors maturation and genotype were not included in this analysis since they were previously shown not to influence the microbiome composition. n.d. = not determined.*

## Discussion

Host-associated microbiomes are important for the nutrition and health of their hosts: plants, animals, and macrofungi are extensively colonized by microorganisms that play key roles in their life cycles (Berg et al., 2014; Bahrndorff et al., 2016; Webster and Thomas, 2016; Pent et al., 2018). Studies on animals and plants have revealed that host identity, genotype, and environmental variables all contribute to shaping the microbial communities colonizing eukaryotic tissues (Bulgarelli et al., 2012; Lundberg et al., 2012; Bouffaud et al., 2014; Hacquard et al., 2015; Glynou et al., 2016), but the relative importance of each factor varies depending on the host and on the type of environment. Fungi also host complex microbial communities that can associate to various fungal structures (i.e., mycorrhizas, mycelium, fruiting bodies) and colonize either the surface of hyphae or the intracellular compartments (Deveau et al., 2018). However, the factors that drive the assembly of these communities are poorly documented. A recent study on the microbiome structure of the epigeous fruiting bodies of the saprophytic fungus *Marasmius oreades* revealed that host genetic differences could be responsible for 25% of bacterial community structure variation (Pent et al., 2018). The authors thus proposed that, similarly to what’s known for animals and plants, host genetics could be an important driver of the structure and function of the microbiome of fungal fruiting bodies (Bulgarelli et al., 2012; Chaston et al., 2016). This was however not the case in this study for *T. aestivum* suggesting that microbiome drivers might thus differ in different fungal species.

### Unexpected Truffle Microbiome Variations Revealed Through Extensive Sampling

The relevance of truffle microbiomes lies in their involvement in aroma production (Splivallo et al., 2015; Splivallo and Ebeler, 2015; Vahdatzadeh et al., 2015) and impact on truffle’s shelf-life/freshness (Rivera et al., 2010). We provide here the first extensive description of the microbiome of the summer black truffle *T. aestivum.* The overall structure of the bacterial communities observed in Swiss and French *T. aestivum* truffles corroborates earlier results obtained from other species of black and white truffles originating from Europe, China, and the United States (Antony-Babu et al., 2014; Barbieri et al., 2016; Benucci and Bonito, 2016; Ye et al., 2018). We confirmed that the *T. aestivum* fruiting body microbiome is characterized by an overall dominance of the α-Proteobacteria mainly affiliated to the *Bradyrhizobium* genus. However, unusual patterns were obtained for about 30% of the fruiting bodies from both Swiss and French truffle orchards. In these cases, microbiomes were dominated by OTUs affiliated to the genera *Pedobacter* (Bacteroidetes), *Polaromonas* (β-Proteobacteria), or *Pseudomonas* (γ-Proteobacteria), and not by α-Proteobacteria. The richness of the communities tended to be reduced to 10–20 OTUs when these genera dominated, suggesting that these particular genera massively invaded the gleba of the fruiting bodies and replaced or competed with *Bradyrhizobium*. By contrast, a few fruiting bodies were characterized by quite diverse and even bacterial communities containing up to 100 different OTUs (e.g., FR_34, CH_55, CH_78). These different microbiome patterns and the occasional preponderance of particular taxa have so far not been reported for any white and black truffle species (Antony-Babu et al., 2014; Vahdatzadeh et al., 2015; Benucci and Bonito, 2016; Ye et al., 2018) but it is in agreement with the discrepancies noticed between studies performed on identical species by different research groups (Barbieri et al., 2016). These differences might be explained by the low numbers of fruiting bodies of diverse truffle species analyzed so far (a few fruiting bodies vs. >50 in our study). Such variability in community composition between fruiting bodies is likely not a specificity of *T. aestivum* truffles, as preliminary results obtained on a large survey of *T. melanosporum* suggest the same trend (Deveau et al., unpublished data).

The ecological function of bacteria colonizing truffle fruiting bodies remains speculative but it has been hypothesized that they might contribute to truffle nutrition as well as aroma variability (Barbieri et al., 2010, 2015; Splivallo and Ebeler, 2015; Vahdatzadeh et al., 2015). It is tempting to speculate that differences in microbial communities might explain variability in aroma documented for *T. aestivum* truffles collected from the same orchard (Splivallo et al., 2012; Molinier et al., 2015). However, aroma variability in *T. aestivum* was linked earlier to truffle genotype, yet genotype did not contribute in the present study to microbial community structuring. This suggests that microorganisms might after all not play a major in the aroma variability of *T. aestivum*. Clearly, this hypothesis will need to be tested in the future, for example, by characterizing the volatile profiles of single major OTUs in the presence of truffle substrate (Splivallo et al., 2015).

The data presented here highlight the importance of three bacterial genera in truffles, namely *Bradyrhizobium*, *Pedobacter*, and *Polaromonas*. Even though the specific functions of these genera in truffles remain to be demonstrated, it has been suggested that *Bradyrhizobium* could be involved in the nutrition of the fruiting bodies since the role of this genus as nitrogen-fixing symbionts is well established in plant roots (Sulieman and Tran, 2014; Coba de la Peña et al., 2018). Nitrogen fixation by *Bradyrhizobium* strains isolated from the white truffle *T. magnatum* has been previously detected (Barbieri et al., 2010), even though several lines of evidence suggest that this might not occur in the black truffle *T. melanosporum* (Barbieri et al., 2016; Le Tacon et al., 2016) where *Bradyrhizobium* strains might be missing the nifH genes involved in nitrogen fixation (Antony-Babu et al., 2014; Deveau et al., unpublished data). This corroborates the recent proposition based on genome comparisons that symbiosis was not the dominant lifestyle of *Bradyrhizobium* but rather on form of specialization (VanInsberghe et al., 2015). *Bradyrhizobium* might also be involved in the production of specific sulfur volatile compounds responsible of truffle aroma perceived by humans (Splivallo et al., 2015). Bacteria of the *Pedobacter* genus have been reported to dominate microbial communities of decomposing fungal mycelium in forest soil and litter (Brabcová et al., 2016). These bacteria regroup generalists that possess a wide array of enzymes allowing degradation of diverse carbon sources. Additionally, some *Pedobacter* produce chitinases to degrade chitin of fungal cell wells. Although no obvious sign of degradation of the fruiting bodies was visible in our samples at the time of harvest, it is tempting to speculate that these bacteria could participate to the degradation of truffle fruiting bodies. Last, the role played by *Polaromonas* in truffles remains elusive. The genus comprises nine commonly occurring species that were originally reported from cold environments. Some members of the *Polaromonas* have the ability to fix nitrogen, hydrogen, and carbon dioxide (Sizova and Panikov, 2007; Hanson et al., 2012), suggesting that they could have similar functions in truffles. Demonstrating the exact function in truffles of these three bacterial genera will be the focus of future work.

### Site-Specific Factors Drive Truffle’s Microbiome Assemblages

Multiple biotic and abiotic factors could drive the composition of the bacterial communities colonizing fruiting bodies of truffles. As the biochemical composition of fruiting bodies strongly changes during several months of maturation of *T. melanosporum* fruiting bodies (Harki et al., 2006), the level of maturity could be an important intrinsic driver of the bacterial communities. Indeed, a correlation was noticed between the bacterial community composition and the level of maturity of fruiting bodies of *T. borchii, T. melanosporum*, and *T. indicum* (Citterio et al., 2001; Antony-Babu et al., 2014; Ye et al., 2018). In contrast, the community composition of the white truffle *T. magnatum* remained stable along the maturation according to Barbieri et al. (2007). Such correlation between maturity degree and the composition of the microbiome was not evidenced in the present study, nor did we observe any link with the abundance of β-Proteobacteria or Bacteroidetes as previously reported by Antony-Babu et al. (2014) in *T. melanosporum*. Whether this is a specificity of *T. aestivum* remains to be determined. A possible reason might be the fact that *T. aestivum*, unlike *T. melanosporum* and other fungi, seems to pass through several lifecycles within a year with no clear seasonality, showing ripe and unripe fruiting bodies uncorrelated with size almost throughout a year (Büntgen et al., 2017). Such asynchrony of maturation might allow to more clearly disentangle maturation from spatial and temporal effects on bacterial communities in truffles. In agreement with this hypothesis, our data highlight a significant contribution of the spatial distance (Swiss orchard) and, to a lesser extent, of the collection year (French orchard) on the community composition of the bacterial communities in fruiting bodies. In addition, since truffle fruiting bodies are likely colonized by bacteria that thrive in the surrounding soil when the embryos are formed (Antony-Babu et al., 2014), such differences could be explained by variations in the bacterial communities of the soil surrounding truffles. Soils properties and climatic conditions likely differed between the two orchards. Similarly, root microbiomes are initially strongly influenced by the composition of the communities of the bulk soil and the environmental factors that influence this “starter” community (Zarraonaindia et al., 2015; Colin et al., 2017). Local pH, nutrients availability, or humidity levels have all been shown to significantly alter soil bacterial community composition (Uroz et al., 2016; Lladó et al., 2017). Although the general physico-chemical properties of soil are likely to be rather homogenous at the scale of a truffle orchard, it is well-known that small-scale heterogeneity exists in soil (Vos et al., 2013). We cannot exclude either that the differences between the factors influencing each orchard’s microbiomes are due to divergent sampling strategies in the two sites: firstly, samples were collected over 2 and 4 years, respectively, and secondly, the two orchards differed in surface area. Altogether this indicates that a better understanding of the interactions between soil microbial communities and truffle embryo at a microscopic scale is required to foresee the process of colonization of truffle fruiting bodies by bacteria.

Taken together, our results provide an unprecedented view of the microbiome associated to the black truffle *T. aestivum*. Microbiomes dominated by either the α-Proteobacteria class, and in some cases the β-Proteobacteria or the Sphingobacteriia classes could be evidenced regardless of geographical origin. The consistent occurrence of those microbes in fruiting bodies from orchards separated by hundreds of kilometers suggests that these bacteria might be highly relevant for truffles ecology and life cycle. Our results also highlight that factors shaping truffle’s microbiomes might differ based on local conditions, but unlike in other fungi, fruiting body maturation and genetic background did not seem to influence the microbiome. Overall, the findings presented here highlight the need to improve our understanding of truffle fruiting body development, of how the truffle microbiome is shaped, and what benefits it provides to truffles (or vice-versa). Complementary studies deploying large sampling efforts and functional studies of main bacterial players of the microbiome will be required to better understand these points.

## Author Contributions

RS designed the experiments and collected the truffle samples. AD and VM characterized the microbiome with input from SU; genotyping by SSR was done by VM, MV, MP, and SE; mating type was determined by VM, MV, MP, SE, and FP. Data was analyzed by MV, RS, AD, and JGMV. AD, JGMV, MV, and RS wrote the manuscript with input from the other co-authors.

## Conflict of Interest Statement

The authors declare that the research was conducted in the absence of any commercial or financial relationships that could be construed as a potential conflict of interest.

## Footnotes

1 http://www.ncbi.nlm.nih.gov/sra
