# Supplementary material for: Orchard Conditions and Fruiting Body Characteristics Drive the Microbiome of the Black Truffle Tuber aestivum
Source: Front Microbiol. 2019 Jun 28;10:1437. doi: 10.3389/fmicb.2019.01437 (PMC6611097; doi:10.3389/fmicb.2019.01437)
Supplement: Supplementary file 1 [file Data_Sheet_1.PDF]

## Supplemental figures & tables to the manuscript

# Orchard conditions and fruiting body characteristics drive the microbiome of the black truffle *Tuber aestivum*

Richard Splivallo*[1], Maryam Vahdatzadeh[1] , Jose G. Maciá-Vicente[2], Virginie Molinier[3,4], Martina Peter[3], Simon Egli[3], Stéphane Uroz[5], Francesco Paolocci[6], Aurélie Deveau*[5]

*1 Institute of Molecular Biosciences, Goethe University Frankfurt, Frankfurt am Main, Germany*

*2 Institute of Ecology, Evolution and Diversity, Goethe University Frankfurt, Frankfurt am Main, Germany*

*3 Swiss Federal Research Institute WSL, Birmensdorf, Switzerland*

*4 UMR 5175 CEFE - CNRS - Université de Montpellier - Université Paul Valéry Montpellier - EPHE - INSERM, Campus CNRS, Montpellier, France*

*5 Institut national de la recherche agronomique (INRA), Unité Mixte de Recherche 1136 INRA-Université de Lorraine, Interactions Arbres/Microorganismes, Centre INRA-Grand Est-Nancy, Champenoux, France.*

*6 National Research Council, Institute of Biosciences and Bioresources - Perugia Division, Perugia, Italy.*

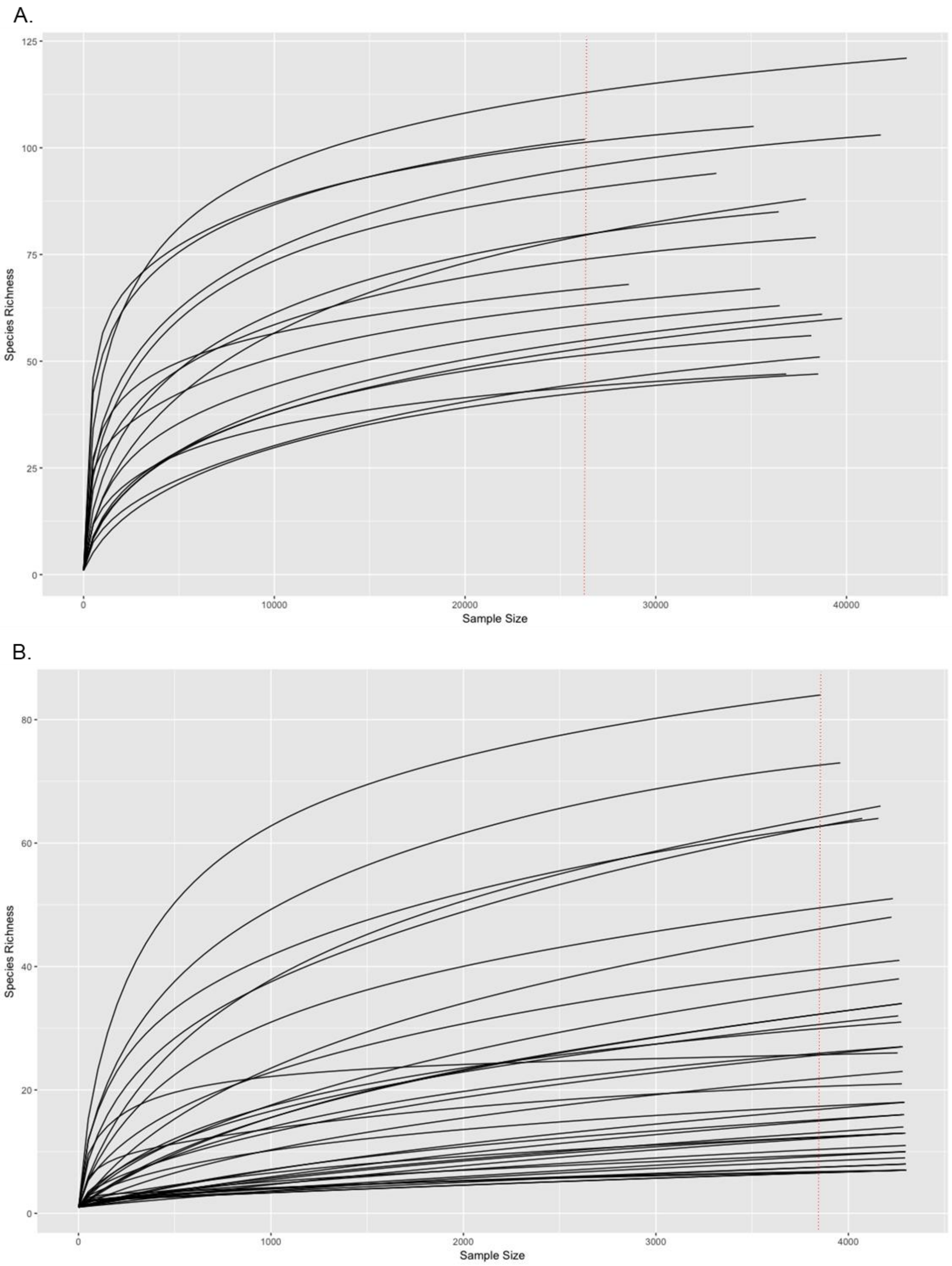


**Supplemental Figure 1. Rarefaction curves**
Rarefaction curves depicting OTUs numbers as a function of sequencing depth for Swiss (A, Illumina sequencing) and French truffle samples (B, 454 sequencing). The red lines indicate the value used for subsampling of the data.

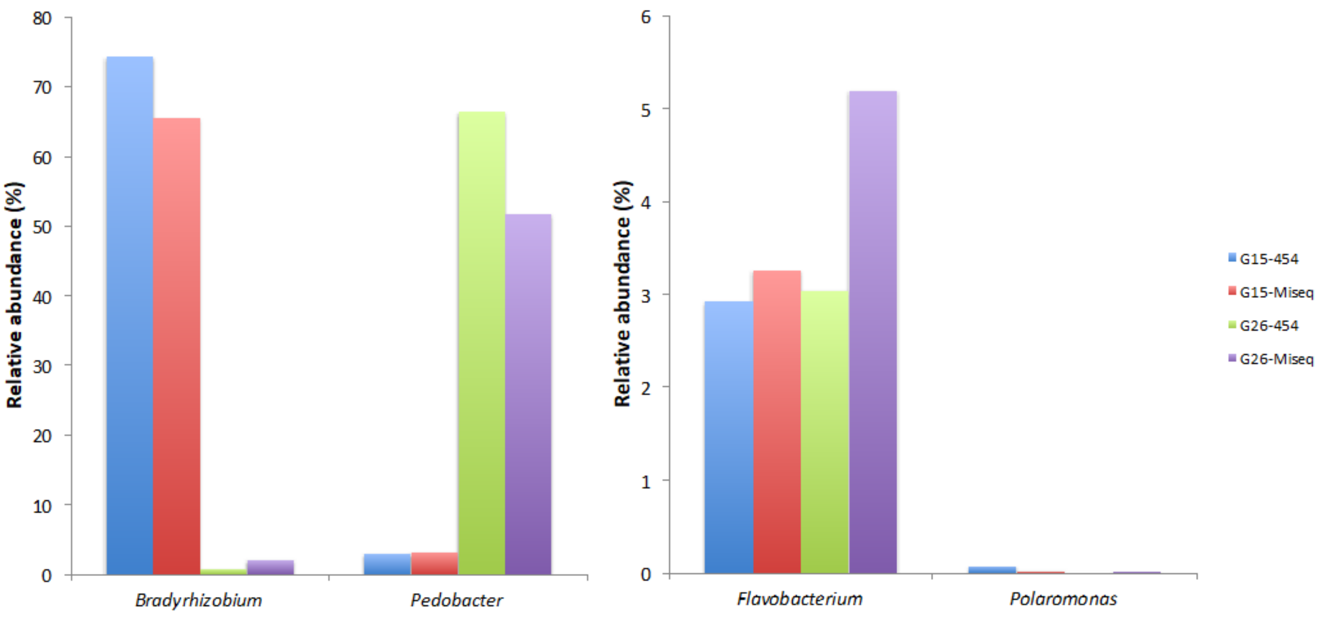


**Supplemental Figure 2. Microbiome of *Tuber melanosporum***
Comparison of the relative abundance of 4 important genera observed in this study as measured by 454 pyrosequecing and Illumina MiSeq of two identical samples. Total DNA of the gleba of two *T. melanosporum* truffles was extracted following the same method described here. Sequencing and data analysis was performed as described in the mat & met section.

**Supplemental Table 1**. List of genera commonly found in truffle ascocarps collected in the French and Swiss orchards and the average relative abundance of reads (± SE).

| Genera | FR [%] | CH [%] |
|---|---|---|
| *Bradyrhizobium* | 65.1 ± 8.8 | 58.6 ± 6.9 |
| *Pseudomonas* | 8.1 ± 3.4 | 3.4 ± 1.4 |
| *Polaromonas* | 5.4 ± 5.0 | 9.2 ± 4.4 |
| *Pedobacter* | 4.3 ± 3.3 | 13.8 ± 4.9 |
| *Flavobacterium* | 2.5 ± 1.2 | 0.81 ± 0.74 |
| *Sphingobacterium* | 1.1 ± 0.7 | 0.18 ± 0.16 |
| *Nocardioides* | 0.52 ± 0.20 | 0.19 ± 0.11 |
| *Chryseobacterium* | 0.43 ± 0.30 | 0.42 ± 0.31 |
| *Rhizobium* | 0.32 ± 0.12 | 0.98 ± 0.43 |
| *Reyranella* | 0.26 ± 0.24 | 0.020 ± 0.010 |
| *Devosia* | 0.21 ± 0.09 | 0.12 ± 0.05 |
| *Shinella* | 0.19 ± 0.05 | 0.074 ± 0.042 |
| *Mycobacterium* | 0.18 ± 0.07 | 0.082 ± 0.036 |
| *Microbacterium* | 0.17 ± 0.10 | 0.063 ± 0.036 |
| *Dyadobacter* | 0.16 ± 0.09 | 0.076 ± 0.030 |
| *Stenotrophomonas* | 0.11 ± 0.08 | 0.56 ± 0.49 |
| *Streptomyces* | 0.11 ± 0.06 | 0.099 ± 0.059 |
| *Clostridium* | 0.100 ± 0.075 | 0.060 ± 0.017 |
| *Opitutus* | 0.096 ± 0.058 | 0.059 ± 0.029 |
| *Acinetobacter* | 0.089 ± 0.019 | 0.057 ± 0.034 |
| *Kribbella* | 0.071 ± 0.042 | 0.037 ± 0.020 |
| *Luteolibacter* | 0.051 ± 0.025 | 0.070 ± 0.040 |
| *Lactococcus* | 0.045 ± 0.008 | 0.003 ± 0.002 |
| *Paenibacillus* | 0.033 ± 0.027 | 0.084 ± 0.058 |
| *Steroidobacter* | 0.026 ± 0.018 | 0.024 ± 0.012 |
| *Providencia* | 0.009 ± 0.004 | 0.001 ± 0.001 |
| *Chitinophaga* | 0.007 ± 0.007 | 0.008 ± 0.005 |
| *Mesorhizobium* | 0.004 ± 0.002 | 0.014 ± 0.008 |
